# The atypical presence of the paternal mitochondrial DNA in somatic tissues of male and female individuals of the blue mussel species *Mytilus galloprovincialis*

**DOI:** 10.1186/1756-0500-3-222

**Published:** 2010-08-06

**Authors:** Eleni Kyriakou, Eleftherios Zouros, George C Rodakis

**Affiliations:** 1 Department of Biochemistry and Molecular Biology, National and Kapodistrian University of Athens, Panepistimioupolis, 15701 Athens, Greece; 2 Department of Biology, University of Crete, 71409 Heraklion, Crete, Greece

## Abstract

**Background:**

In animals mtDNA inheritance is maternal except in certain molluscan bivalve species which have a paternally inherited mitochondrial genome (genome M) along with the standard maternal one (genome F). Normally, the paternal genome occurs in the male gonad, but it can be often found, as a minority, in somatic tissues of males and females. This may happen in two ways. One is through "sperm mtDNA leakage" into somatic tissues, a deviation from the normal situation in which the sperm mtDNA vanishes in females or ends up exclusively in the germ line of males. The other is through "egg heteroplasmy", when the egg contains, in small quantities, the paternal genome in addition to maternal genome.

**Findings:**

To test the two hypotheses, we compared the sequences of one of the most variable domains of the M molecule in a somatic tissue (foot) and in the sperm of ten male and in the foot of ten female individuals of *M. galloprovincialis*. Presence of the M genome was rarer in the foot of females than males. The M genome in the sperm and in the foot of males was identical.

**Conclusions:**

Given that the surveyed region differs from individual to individual, the identity of the M genome in the foot and the sperm of males supports strongly the hypothesis that, at least for the tissue examined, the presence of the M genome is due to sperm mtDNA leakage.

## Background

Contrary to the general rule of maternal inheritance of mitochondrial DNA (mtDNA) in metazoans [1-3], in some bivalve mollusks we observe the phenomenon of Doubly Uniparental Inheritance (DUI), according to which a maternally inherited mitochondrial genome (the F-type mtDNA) is present in the eggs and the somatic tissues of female and male individuals, whereas a different paternally inherited mitochondrial genome (the M-type mtDNA) appears in the male germ line [4-7]. The DUI is maintained through the transmission of the F-type from the mother to both female and male offspring and the transmission of the M-type from the father to sons. In *Mytilus *species, where DUI has been studied extensively, the sperm carries on average five mitochondria [8] that contain only the paternally inherited M-type mitochondrial DNA [9]. In contrast, an unfertilized egg contains many tens of thousands, perhaps several hundreds of thousands, of mitochondria [10] that contain the F-type molecule. After fertilization, both male and female embryos receive paternal mitochondria through the sperm [11]. In female embryos, the five sperm mitochondria disperse randomly among cells after zygote division, and are stochastically lost during development as a tiny minority in the pool of maternal mitochondria. On the contrary, in male embryos, sperm mitochondria tend to stay together as an aggregation in the same cell during successive divisions. This is the cell that is destined to produce, among others, the germ line in the following developmental stages [12-14]. However, the M-type may also be found in small quantities in somatic tissues of female and male individuals [15-17]. According to Garrido-Ramos et al. [16], the M molecule is occasionally found in the foot, gill, adductor, digestive gland and mantle of male individuals of the species *Mytilus edulis*, beside its normal appearance in the gonad of all males. Moreover, the M-molecule also appears in the somatic tissues of some female individuals [16,18].

This report is a further investigation of this atypical presence of the paternal mtDNA in male or female somatic tissues. Two hypotheses can be proposed for this phenomenon. The first - referred to herein as "sperm mtDNA leakage" - is that the M-type mtDNA of somatic tissues originates from the sperm that produced the individual. In males "leakage" of the M-type mtDNA in somatic tissues could occur if some of the five sperm mitochondria (or even one) detached from the aggregation mentioned above and ended up in embryonic cells that will later form somatic tissues [13]. In females, the same result could be obtained if some (or even one) of the sperm mitochondria that are randomly scattered among cells are not stochastically lost during successive cell divisions in the face of the huge excess of egg mitochondria, but established themselves through proliferation, in some primordial somatic cells. The second hypothesis - referred to herein as "egg heteroplasmy" - is that the egg may itself contain a small amount of the M genome, in the same way that was just described for a female's somatic tissue. This M-type mtDNA will henceforth behave as a maternal mtDNA, i.e. it will be dispersed in the somatic cells of the new embryo, and will not be diverted into the gonad, should the embryo develop into a male individual. That this may be a way through which female and male somatic tissues may become heteroplasmic for the F and M genomes is supported by the work of Obata et al. [18,19], who reported the presence of heteroplasmic eggs in *M. galloprovincialis*. Theologidis [20] discussed the somatic tissue heteroplasmy in species with DUI and suggested that a combination of the two routes in the same individual may even lead to triplasmy - if there is sperm mtDNA leakage in an embryo that resulted from a heteroplasmic egg. In principle, a heteroplasmic individual could also result from a heteroplasmic sperm. However, this third possibility appears unlikely given the observation of Venetis et al. [9], who found that a son's sperm contained only the son's paternal mtDNA.

The two routes through which a somatic tissue may become heteroplasmic for the maternal and paternal mtDNA genomes lead to a different prediction about what one may find if one could compare the M-type present in the sperm of a male with that found in its somatic cells. The first route predicts that sperm and somatic M-type mtDNA will be identical, given that both can be traced to the sperm that produced the particular male. The second route leaves open the possibility that the two types would be different, given that the M-type in the sperm and the M-type in the somatic tissue originated from different males (the first from the male's father and the second from the male's maternal grandfather). To test this possibility, we compared the sequence of a part of the M molecule from the foot and the sperm of *M. galloprovincialis *males. The mitochondrial genome of *Mytilus *consists of the "core" that contains all protein, tRNA and rRNA genes (and a few non-coding sequences of small length) and the control region (CR), which is further divided in three parts, the first variable domain (VD1), the conserved domain (CD) and the second variable domain (VD2) [21]. The part that we selected was the first variable domain (VD1). This region is the most variable part of the mussel mtDNA, both in the F and the M genome [21,22]. We chose the foot as the somatic tissue to be examined because in the mussel's body, the foot does not come in direct contact with the gonad, unlike other often-used tissues, such as the gill or the mantle and can, for this reason, be excised from the animal with minimal risk of contamination from gonadal tissue that carries large quantities of the M-type molecule. The rationale of the study was that if the sequences recovered from the foot and the sperm of a male were different we could conclude with a high degree of certainty that this male's heteroplasmy resulted according to the second mechanism. If the sequences were found to be the same, then the first mechanism is most likely, given that the probability that the male's paternal and grand-paternal M-type mtDNA had exactly the same sequence in the examined mtDNA region would be small.

## Methods

### Sampling and DNA extraction

Adult mussels (*Mytilus galloprovincialis*) were kindly provided by 'Poseidon Co.', a mussel growing operation in New Peramos (Saronikos Gulf, app. 50 km West from Athens, Greece) during the months November and December of 2008, when the gonads were reproductively mature and ready for spawning. Mussels were placed in individual containers filled with seawater at 6-8°C and one day later transferred to room temperature (22-25°C) to induce spawning by thermal shock. Salt shock (injection of ~1 ml of a KCl solution between the shells) was also used for spawning induction of individuals that did not spawn after the initial shock. The individuals were sexed by microscopic examination of the released gametes. To eliminate the possibility of contamination of DNA preparations from sperm with DNA from cells of somatic origin, spermatozoa were forced to swim through a solution of Percoll as described by Venetis et al. [9]. To avoid contamination of foot tissues with mtDNA from the gonad, the individual was boiled after spawning for 1 min prior to dissection. This procedure hardens the tissues of the individual (including the gonad) and allows their separation, without causing any damage to the DNA. This procedure was followed for DNA extraction from the foot of males and females. Total DNA was extracted according to Douris et al. [23].

### PCR amplification

Extracted DNA from sperm and foot was used as template to amplify the first variable domain (VD1) of the control region (CR) [21]. VD1 was chosen because of its high degree of divergence between F and M genomes and also because of its hypervariability among M and among F genomes from different individuals. The forward primer 16M-Sl-F 5'-GTG YCC TAG AGG YGT CGA CGC YTC TAA G-3' [22] and reverse primer muDLR 5'-CTC TGA CAA ATG CTT ATY AGC TG-3' [22] were used to amplify the fragment between positions #17448 and #542 of the complete circular M-type genome of *M. galloprovincialis *(accession no: AY363687). These primers are M-specific, i.e., they do not amplify from the F genome. The amplified fragment contains, from upstream (5') to downstream (3'), 165 bp of the 16S-rRNA gene, the entire VD1, and 26 bp of the conserved domain (CD) of the CR. Forward primer ssFdl1: 5'-GGT GAT AGG TTG TTA AGY GTG G-3' [22] and the reverse primer ssFdl2: 5'-CAC CGT CRC CTT CTC CWC CC-3' [22] were used to amplify the fragment between positions #28 and #214 bp of the complete circular F-type genome of *M. galloprovincialis *(accession no: AY497292). These primers are F specific, i.e. they do not amplify from the M genome. The amplified fragment contains 187 bp of VD1. All samples (foot from both males and females and sperm from males) were tested for the presence of the M and the F genome.

In addition to the F and M genomes, *M. galloprovincialis *populations may contain, at substantial frequencies, a third class of genomes whose control region consists of sequences from the F and the M genome [24,25]. These recombinant genomes are paternally inherited, even though this has not been demonstrated for any one of them. One such genome, the genome C [24], which is paternally inherited, occurs in low frequency in the area from which our sample was taken [9,24]. We used the primers S35-vd1M-f1: 5'-GCT TGT AAM AGA ART AGT CSC CTC-3'and QssMdl-2: 5'- CTG GTA TAC ACA CGY TAC ACA CC-3' [24] to check for the presence of this genome in the samples we examined. These primers amplify only fractions of C genome [positions 1257-1967 bp, 2045-2755 bp and 1257-2755 bp in the *M. galloprovincialis *complete C genome (GenBank Acc. No DQ399833)], thus producing a profile that distinguishes this genome from the F and the M genomes.

Promega *Taq *polymerase was used in all polymerase chain reactions (PCR). PCR amplifications were carried out in 25-50- μl reaction volumes containing ~50-100 ng of template DNA, 0.5-1 μM of each primer, 200 μM of each dNTP, 2-2.5 mM MgCl_2_, and 0.625-1.25 units *Taq *polymerase in the buffer supplied by the company. The reactions were heated initially at 94°C for 2 min and then incubated at 94°C for 20-35 sec, 56-58°C (depending on the Tm of the primer pair) for 30-35 sec and 72°C for 13-110 sec (depending on the length of the amplified product) for 35-40 cycles and 72°C for 5 min for a final extension. The amplified products were visualized on a 1% agarose gel.

### Cloning and Sequencing

DNA from PCR products was recovered using the PureLink Quick Gel Extraction Kit (Invitrogen) and the NucleoSpin Extract II PCR purification kit (Macherey-Nagel) according to the company's instructions. The purified PCR products were sequenced directly or after cloning from both directions using a commercial outlet (Macrogen, South Korea). DNA fragments were cloned in pGEM-T vector (Promega) or pTZ57R/T vector (Fermentas) following the procedure provided by the supplier in *Escherichia coli *DH5α cells. Recombinant plasmid DNA from one positive clone from each transformation was isolated using standard procedure and sequenced commercially as mentioned above.

The DNA sample of the foot of one male was accidentally damaged, thus this male was not entered in the sequence comparison. To have a comparable number of M sequences from both genders we returned to the females that did not spawn and amplified for the M genome in foot preparations. We got 12 positive reactions out of the 70 females that we screened for this purpose and sequenced the product from eight of them taken at random. This gave us a set of 30 M-type sequences for comparison: 10 from the foot of 10 different females, 10 from the foot of 10 different males and 10 from the sperm of these same males. All sequences obtained were deposited in GenBank under the Accession Numbers HM027601-HM027630.

### Sequence analysis

The length of the obtained sequences varied from 658 to 693 bp. This is due to several indels at a poly(A) stretch that occur in the first variable domain (VD1) of the control region (CR) of the M genome, specifically between nucleotide positions 207 and 232 (reference sequence AY363687 of the first complete M-type sequence reported [22]). This stretch, whose length varies from 23 to 38 bp in the complete sequences of M genome of *M. galloprovincialis/M. edulis *currently available in the literature [22,26-28], causes DNA strand slippages and mismatches during PCR and sequencing steps. As a result the number of bases downstream nucleotide position 207 cannot be scored. The part of sequence that follows the A-rich region could be read unambiguously from the reverse DNA strand. To avoid PCR-induced differences among sequences, we excluded the A-rich region from all 30 compared sequences. In four cases (m12ft, m12sp, f7ft and f13ft, [see Additional file 1]) sequence uncertainties were resolved by cloning and sequencing the PCR-product.

Sequences were aligned on the basis of nucleotide similarity using the default parameters of ClustalX v. 1.83 [29], followed by minor corrections to maximize their similarity. Number of differences at the nucleotide level was calculated by pairwise comparison using MEGA version 3.1 [30]. Gaps/Missing Data parameter was set at "Pairwise Deletions".

## Results and Discussion

### Presence of different mtDNA types

Out of 424 individuals of *Mytilus galloprovincialis *36 spawned: 17 males and 19 females. Total DNA was isolated from the foot and sperm of these males and from the foot of females. The PCR assay for the C-genome was negative in all samples. All foot samples, from either female or male individuals, were positive for the F-genome, but no sperm sample tested positive for this genome. The M-specific test was positive in two female foot preparations out of 19 (or 10% of cases) and in 11 male foot preparations out of 17 (or 65%). The frequency of occurrence of the M genome in the foot tissue is statistically higher in males (chi-square 11.885, D.F. = 1, P = 0.0006). This result agrees with that by Garrido-Ramos et al. [16], who observed that the M genome was more frequently found in the foot of males than females of *M. edulis*. Further comparison of the two studies is not possible because of the different ways of detection of the M genome they used.

### Sequence comparison

To obtain an equal number of M-positive foot samples from both genders we extracted DNA from the foot of females that did not spawn (see Methods). The complete set of 30 sequences we obtained (ten foot sequences from ten females and ten foot and ten sperm sequences from ten males) are given in Additional file 1. The complete sequence of VD1 (see Additional file 1), including the A-rich region that was excluded from the sequence comparison. In contrast, only the variable positions of the *16S-rRNA *and the CD fragments are shown. The most striking observation is that in each of the ten males the sequence from the foot and the sequence from the sperm were identical. Among the 30 sequences one was encountered 5 times (f7, m9ft, m9sp, m17ft, m17sp), one 4 times (f80, f81, m16ft, m16sp), one tree times (f1, f2, f11), 7 twice each in a different male and 4 once each in a different female. This provides a rough estimate, the best we can have on the basis of our sample size, of haplotype frequencies under the hypothesis that the foot and sperm haplotype in a male are independently derived. For the hypothesis of egg heteroplasmy to be compatible with our data we would require that the male's father and the male's maternal grand-father happened to have the same sequence. The probability, p, that this may occur for any single male is p = (5/30)^2 ^+ (4/30)^2 ^+ (3/30)^2 ^+ 7(2/30)^2 ^+ 4(1/30)^2 ^= 0.091. The probability, P, that this will happen for all ten males is P = (0.091)^10 ^= 4 × 10^-11^, an extremely small number. We may confidently conclude that the presence of the M genome in the foot of males is due to sperm mtDNA leakage. On the basis of this conclusion, the set of independently derived sequences is reduced to 20. The new probability for obtaining the same sequence in two draws is h = 11(1/20)^2 ^+ 3(3/20)^2 ^= 0.095. This is not very different from the one obtained when the two sequences in a male were assumed to be independently derived, and this is due to the high diversity of the VD1 region.

For females we cannot have direct evidence that the M genome found in the foot is that of their father because we do not have an independent way of knowing what the father's M genome was, as we do have in males. This could be done only in females with a known male parent. Even though several studies of DUI have used pair-matings [7,31,32], none of these compared the M genome of the male parent with that found in the somatic tissues of females. Our results from females do not provide information about the origin of the M genome in somatic tissues but suggest that the M sequences found among females are no less diverse than those found in males, in agreement with expectation. Among the ten females we encountered one sequence three times (f1, f2, f11), another sequence twice (f80, f81) and five singletons (h = 0.02). Assuming that the two sequences in a male have the same origin, we encountered one sequence twice (m9 and m17) and 8 singletons (h = 0.014).

Our results can be discussed in the context of the studies by Cao et al. [12], Obata & Kumaru [14], Cogswell et al. [13] and Kenchington et al. [33]. In these studies sperm of *Mytilus edulis *(in the first, third and fourth study) and of *Mytilus galloprovincialis *(in the second study) was treated with MitoTracker Green FM, a fluorescent stain that binds to the outer surface of mitochondria, and used to fertilize eggs. The sperm mitochondria were observed microscopically in the embryo up to the 4-cell stage (and, occasionally, up to the 8-cell stage). The eggs used in these experiments were derived from mothers that were known from previous studies to produce daughters either exclusively or in very high frequency (daughter-biased mothers) or from mothers that produced sons in high frequency (son-biased mothers). The researchers found that in embryos from daughter-biased mothers the sperm mitochondria dispersed randomly in both blastomeres at the 2-cell stage or in all four blastomeres at the 4-cell stage and that the distribution pattern was random. Thus, in female embryos, the sperm mitochondria appear to be randomly mixed with the egg mitochondria, which vastly outnumber them, and are either entirely lost or become barely detectable minorities in the somatic tissues of adult females. In contrast, in embryos from son-biased mothers the five sperm mitochondria formed a condensed mass that was found in the larger cell resulting from the first or second egg division. The researchers suggest that this is a developmental mechanism through which the sperm mtDNA is delivered in the male's primordial germ cells. Thus in males sperm mitochondria appear to follow a regimented developmental process, the result of which is that the male gonad becomes mainly or exclusively occupied by the sperm mtDNA (M-type), whereas the somatic tissues contain mainly the egg mtDNA (F-type). We say mainly, not wholly, because according to the results of several studies [15-19] the M-type is also found in somatic tissues of some males.

Our study is the first to compare the M genome that is always found in the gonad of a male and the M genome that is occasionally found in the somatic tissues of the same male (but see [34]) and leads to the conclusion that the M molecule found in the foot of these individuals comes from leakage of sperm mtDNA into somatic tissues. This leakage may occur if one or more of the approximately five sperm mitochondria that enter the egg after fertilization fail to become part of the aggregate that is formed by the sperm mitochondria and, as a result, ends up in cells that will form somatic tissues. Cao et al. [12], Cogswell et al. [13] and Kenchington et al. [33] have indeed observed repeatedly cases of "orphan" sperm mitochondria, i.e., mitochondria that were not part of the aggregate and segregated independently from it. Our results do not exclude the hypothesis that some cases of somatic heteroplasmy for the M genome in species with the DUI system may result from heteroplasmic eggs, i.e., eggs that contain a minority of M mtDNA genomes along with the vast majority of F genomes. This hypothesis is supported by the work of Obata et al. [18,19], who reported a high incidence of egg heteroplasmy. Further evidence for this comes from the work of Theologidis [20], who has observed several cases of triplasmy in male and female tissues, i.e., presence of F, M and C genomes. That this situation may have arisen through sperm heteroplasmy (a sperm that contained the M and the C genomes) appears unlikely in view of the results of Venetis et al. [9] who failed to detect in the sperm of 35 males any other mtDNA except the paternal one. We note that the mechanism of egg heteroplasmy, if it occurs, requires that the female that produces the heteroplasmic egg must have received its M genome from its father or from a more remote ancestral male. This means that sperm mtDNA leakage is the ultimate cause of the presence of the M genome in either male or female somatic tissues.

## Competing interests

The authors declare that they have no competing interests.

## Authors' contributions

EK carried out the experiments and drafted the manuscript. EZ and GCR supervised the work, participated in the experimental design and helped in manuscript preparation. All authors contributed to data interpretation and analysis, and have read and approved the manuscript.

## Supplementary Material

Additional file 1 **The M-type sequences found in the foot (m_ft) and the sperm (m_sp) of ten males and the foot of ten females (f_ft)**. The sequences include the full length of VD1 and only the nucleotide of the variable sites of the amplified parts of the *16S-rRNA *gene and CD. The A-rich stretch of VD1 is highlighted. Dots imply nucleotide identity with the sequence given at the top and dashes indicate deletions. The alignment position numbers are given above the sequence. Click here for file
