# Supplementary material for: The atypical presence of the paternal mitochondrial DNA in somatic tissues of male and female individuals of the blue mussel species Mytilus galloprovincialis
Source: BMC Res Notes. 2010 Aug 6;3:222. doi: 10.1186/1756-0500-3-222 (PMC2924344; doi:10.1186/1756-0500-3-222)
Supplement: Additional file 1 — The M-type sequences found in the foot (m_ft) and the sperm (m_sp) of ten males and the foot of ten females (f_ft). The sequences include the full length of VD1 and only the nucleotide of the variable sites of the amplified parts of the 16S-rRNA gene and CD. The A-rich stretch of VD1 is highlighted. Dots imply nucleotide identity with the sequence given at the top and dashes indicate deletions. The alignment position numbers are given above the sequence. [file 1756-0500-3-222-S1.PDF]

```
... 16S-rRNA (1-165)->| |-> VD1 of CR (alignment positions 166-673)
           111111111111 1   1         1         1         2         2         2         2         2         2         2         2         2         2         3
       2558000112334446 6   7         8         9         0         1         2         3         4         5         6         7         8         9         0
       3060345027012672 6789012345678901234567890123456789012345678901234567890123456789012345678901234567890123456789012345678901234567890123456789012345678

m6ft   AAGTCCCCAATTTGTA CCTTTTATGTAAGTGAGGTTGGCTATAAGTGTACTAGACTT--TACAGGAATGTACGCAGATAGTTGGAGATAGT-CTCACCTTGAAAAAGAG--TGTTGTATCGCGTATATGAGAGGCCTACCTGAACAACAGAGTAATCC
m6sp   ................ .........................................--................................-.................--...............................................
m8ft   ............C... ...................C.....................--..T......A......................-.................--..C............................................
m8sp   ............C... ...................C.....................--..T......A......................-.................--..C............................................
m9ft   ............C... ....................................T....--.........A.................C....T-.G..............--............C.....G.............G..............
m9sp   ............C... ....................................T....--.........A.................C....T-.G..............--............C.....G.............G..............
m10ft  G.A......TG.CTCG ......G.....A-.......T..........T........-C..T...G.G.GG.T...T..A..A..AG..ACC.-..........-..-.CC...C..-.T.A.......G.G..TT.GTTCA.....T.A.C....TT
m10sp  G.A......TG.CTCG ......G.....A-.......T..........T........-C..T...G.G.GG.T...T..A..A..AG..ACC.-..........-..-.CC...C..-.T.A.......G.G..TT.GTTCA.....T.A.C....TT
m12ft  ............C... .........................................--..T......A....................A.-.................--..C............................................
m12sp  ............C... .........................................--..T......A....................A.-.................--..C............................................
m13ft  ............C... .........................................--.........A.G....................-.................--............................T...........A......
m13sp  ............C... .........................................--.........A.G....................-.................--............................T...........A......
m14ft  ............C... ...............G.........................--.........A......................-.................--...............................................
m14sp  ............C... ...............G.........................--.........A......................-.................--...............................................
m15ft  .G..........C... .........................................--.........A......................-......C..........--.......................T.......................
m15sp  .G..........C... .........................................--.........A......................-......C..........--.......................T.......................
m16ft  ...........CC... ......................................T..--.........A.....................--------------------------............A.............................
m16sp  ...........CC... ......................................T..--.........A.....................--------------------------............A.............................
m17ft  ............C... ....................................T....--.........A.................C....T-.G..............--............C.....G.............G..............
m17sp  ............C... ....................................T....--.........A.................C....T-.G..............--............C.....G.............G..............
f1ft   ...C...TG...C... ..........................-----..---.....--.........A.............-----------................--....................A..........................
f2ft   ...C...TG...C... ..........................-----..---.....--.........A.............-----------................--....................A..........................
f7ft   ............C... ....................................T....--.........A.................C....T-.G..............--............C.....G.............G..............
f8ft   ...C...TG...C... ..........................-----..---..G..--.........A.............-----------........G.......--...............................................
f11ft  ...C...TG...C... ..........................-----..---.....--.........A.............-----------................--....................A..........................
f13ft  .......TG...C... ..........................-----..---.....--.........A.............-----------................--....................A..........................
f80ft  ...........CC... ......................................T..--.........A.....................--------------------------............A.............................
f81ft  ...........CC... ......................................T..--.........A.....................--------------------------............A.............................
f103ft ............C..G .........................................--..T......A......................-.................--..C............................................
f112ft G.A.T.G..TA.CTCG ............A.-.................T.....-..CC......G.G.GG.C...T..A..A..AG..A.T............-..-.CC....T.-.T.A.........G...T.GTTCA....T..A.C....T.
```

```
        3         3         3         3         3         3         3         3         3         4         4         4         4         4         4         4
        1         2         3         4         5         6         7         8         9         0         1         2         3         4         5         6
       901234567890123456789012345678901234567890123456789012345678901234567890123456789012345678901234567890123456789012345678901234567890123456789012345678901234567

m6ft   AGGGG-AAAGAGGTGCGAGTCTCGTAAAAA-TAGGAATAAAGCTACCTAAAAAATATGGTGTGTAACGTGTGTATACAAGTATACGCAAAAAAAAAAAAAAAAAAAAAAAAAAA-----------CCGTAAAATGTTTAGGAATAAGGTGTTTCTACAC
m6sp   .....-........................-...................................................................................-----------..................................
m8ft   .....-........................-...................................................................................A----------..................................
m8sp   .....-........................-...................................................................................A----------..................................
m9ft   .....-........................-.T.A..............................................................................------------.............G..............TC....
m9sp   .....-........................-.T.A..............................................................................------------.............G..............TC....
m10ft  ...A.G...A.-...T......TT.....GA..AA..C..G......C.................G................................................TA---------..A....-.....GAAG.......A....A....
m10sp  ...A.G...A.-...T......TT.....GA..AA..C..G......C.................G................................................TA---------..A....-.....GAAG.......A....A....
m12ft  .....-........................-...................................................................................-----------..................................
m12sp  .....-........................-...................................................................................-----------..................................
m13ft  .....G........................-...................................................................................AAAAAAAAAAA..................................
m13sp  .....G........................-...................................................................................AAAAAAAAAAA..................................
m14ft  .....-..G.....................-..................................................................................------------..................................
m14sp  .....-..G.....................-..................................................................................------------..................................
m15ft  .....-.........T..............-...................................................................................AAAAAAAAAAA..................................
m15sp  .....-.........T..............-...................................................................................AAAAAAAAAAA..................................
m16ft  .....-........................-........................C..........................................................AA---------...............................T..
m16sp  .....-........................-........................C..........................................................AA---------...............................T..
```

```
m17ft   .....-........................-.T.A...............................................................................A----------.............G..............TC....
m17sp   .....-........................-.T.A...............................................................................A----------.............G..............TC....
f1ft    .....-........................-...................................T.........T..................................--------------.............G....................
f2ft    .....-........................-...................................T.........T..................................--------------.............G....................
f7ft    .....-........................-.T.A...............................................................................A----------.............G..............TC....
f8ft    .....-........................-...................................T.........T.................................---------------.............G..............T.....
f11ft   .....-........................-...................................T.........T..................................--------------.............G....................
f13ft   .....-........................-...................................G.........T...................................-------------.............G....................
f80ft   .....-........................-........................C..........................................................A----------...............................T..
f81ft   .....-........................-........................C..........................................................-----------...............................T..
f103ft  .....-........................-..................................................................................------------..................................
f112ft  .....G-C..............TT......-...A.....G......C.................G...............................................TA----------..A....-......AA..................

          4         4         4         5         5         5         5         5         5         5         5         5         5         6         6         6
          7         8         9         0         1         2         3         4         5         6         7         8         9         0         1         2
        890123456789012345678901234567890123456789012345678901234567890123456789012345678901234567890123456789012345678901234567890123456789012345678901234567890123456
m6ft    GCTTAGATTCCTTGCCATTGCCTGTAACAGAAGCAATCGCCTCGGTTCCTCTGTTTTTTTACACGTAAAAGTCCCCTGTTGACACACATGGGAGCCGCCTTATTAAAATAACTTATAATCTAAGTGAAAGCACACCTAATTAGTTTTTATTAGGCATTT
m6sp    ...............................................................................................................................................................
m8ft    .........................G.............................................................................................T.......................................
m8sp    .........................G.............................................................................................T.......................................
m9ft    ...........................................T...................................................................................................................
m9sp    ...........................................T...................................................................................................................
m10ft   .....AC....C.....C...T...........T.G.......T..C..C...........T...........TA......G....T...A....TA.T...C.G.............A.....C....................C.............
m10sp   .....AC....C.....C...T...........T.G.......T..C..C...........T...........TA......G....T...A....TA.T...C.G.............A.....C....................C.............
m12ft   .........................G.....................................................................................................................................
m12sp   .........................G.....................................................................................................................................
m13ft   ..........................................................................T....................................................................................
m13sp   ..........................................................................T....................................................................................
m14ft   ................................................................................................................C..............................................
m14sp   ................................................................................................................C..............................................
m15ft   ...............................................................................................................................................................
m15sp   ...............................................................................................................................................................
m16ft   ...................................G.......T.....C.............................................................................................................
m16sp   ...................................G.......T.....C.............................................................................................................
m17ft   ...........................................T...................................................................................................................
m17sp   ...........................................T...................................................................................................................
f1ft    .......C.................G.................A.....C.................................G...................................A.......................................
f2ft    .......C.................G.................A.....C.................................G...................................A.......................................
f7ft    ...........................................T...................................................................................................................
f8ft    .......C.................G.................A.......................................G...................................A.......................................
f11ft   .......C.................G.................A.....C.................................G...................................A.......................................
f13ft   .......C.................G.................A.....C.................................G...................................A.......................................
f80ft   ...................................G.......T.....C.............................................................................................................
f81ft   ...................................G.......T.....C.............................................................................................................
f103ft  .........................G.....................................................................................................................................
f112ft  .....AC....C.....C...T...........T.G.......T..C..C...........T...........TA......G....T...A....TA.T...C...............A.....C.................A..C.............
```

```
                                                   ->| |-> CD of CR (674-699)...
          6         6         6         6         6    6
          3         4         5         6         7    8
       78901234567890123456789012345678901234567890123 5
m6ft   ATACTTTATTTAAAATTTAGGCCCATATGTCACAGATACCTAGCCAT G
m6sp   ............................................... .
m8ft   ............................................... .
m8sp   ............................................... .
m9ft   ...G......C.......G.................C.......... .
m9sp   ...G......C.......G.................C.......... .
m10ft  ...A......C....A..G.....G.....T......C......... .
m10sp  ...A......C....A..G.....G.....T......C......... .
m12ft  ............................................... .
m12sp  ............................................... .
m13ft  ............................................... .
m13sp  ............................................... .
m14ft  ...G........................................... T
m14sp  ...G........................................... T
m15ft  ........................G.....GT............... .
m15sp  ........................G.....GT............... .
m16ft  ...G...G....................................... .
m16sp  ...G...G....................................... .
m17ft  ...G......C.......G.................C.......... .
m17sp  ...G......C.......G.................C.......... .
---------------------------------------------------------
f1ft   ...G......C.................................... .
f2ft   ...G......C.................................... .
f7ft   ...G......C.......G.................C.......... .
f8ft   ...G......C.................................... .
f11ft  ...G......C.................................... .
f13ft  ...G......C.................................... .
f80ft  ...G...G....................................... .
f81ft  ...G...G....................................... .
f103ft ............................................... .
f112ft ...T......C....A..G.....G............T......... .
```

Additional file

The M-type sequences found in the foot (m_ft) and the sperm (m_sp) of ten males and the foot of ten females (f_ft). The sequences include the full length of VD1 and only the nucleotide of the variable sites of the amplified parts of the 16S gene and CD. The A-rich stretch of VD1 is highlighted. Dots imply nucleotide identity with the sequence given at the top and dashes indicate deletions. The alignment position numbers are given above the sequence.
